# 2952. Impacts of an evidence-based, pre-populated order panel on antimicrobial prescribing trends in ambulatory patients with community-acquired pneumonia

**DOI:** 10.1093/ofid/ofad500.191

**Published:** 2023-11-27

**Authors:** Ryan W W Stevens, Kellie Arensman Hannan, Caitlin C Schanz, Paschalis Vergidis, Benjamin Anderson, Ross Dierkhising, Kirstin Kooda, Abinash Virk, Kelsey L Jensen

**Affiliations:** Mayo Clinic, Rochester, MN; Mayo Clinic Health System, Mankato, Minnesota; Mayo Clinic - Rochester, Rochester, Minnesota; Mayo Clinic, Rochester, MN; Mayo Clinic - Rochester, Rochester, Minnesota; Mayo Clinic, Rochester, MN; Mayo Clinic - Rochester, Rochester, Minnesota; Mayo Clinic, Rochester, MN; Mayo Clinic Health System - Southeast Minnesota, Osage, Iowa

## Abstract

**Background:**

Optimizing antimicrobial regimens for outpatients with community-acquired pneumonia (CAP) represents an area of opportunity for ambulatory antimicrobial stewardship programs. A pre-populated, antibiotic order panel for outpatient respiratory syndromes was implemented in the Mayo Clinic Enterprise on 1/4/2021. For CAP, it provides national guideline adherent regimens (i.e., selection, dosing, and durations) by patient comorbidity and beta-lactam allergy status. We aimed to assess impacts of panel use on antibiotic prescribing in outpatients with CAP.

**Methods:**

This retrospective review included Mayo Clinic outpatient encounters from Minnesota in 2021 and 2022. Eligible encounters used a CAP-related primary ICD-10 code, conveyed intention to treat CAP in encounter notes, and were performed by primary care, emergency department, or urgent care clinicians who managed ≥1 CAP patient with the panel and ≥1 without the panel during the study period. Encounters were excluded if patients were already receiving antibiotics at the time of encounter. The primary outcome was adherence (drug and duration) to guideline recommendations. Secondary outcomes included utilization rate by agent and therapy duration. Outcomes were analyzed with Kruskal-Wallis rank sum and Pearson’s chi-squared tests (α level = 0.05).

**Results:**

352 encounters (134 panel vs. 218 non-panel) were included. Prescribing was guideline adherent in 61.9% and 29.4% (p< 0.01) of panel and non-panel encounters, respectively. The most common reason for guideline non-adherence during panel use was inappropriate drug selection (n = 45/51, 88.2%). This was predominantly driven by use of the incorrect section of the treatment panel based on patient comorbidity status (n = 30/45, 66.7%). Individual antibiotic utilization rates differed between panel and non-panel cohorts (Table 1), with higher rates of amoxicillin and amoxicillin/clavulanate use and lower rates of azithromycin and doxycycline use in the panel cohort. Durations >5 days were more common in the non-panel as compared to the panel cohort (34.4% vs. 7.5%, p < 0.01).

**Figure:**
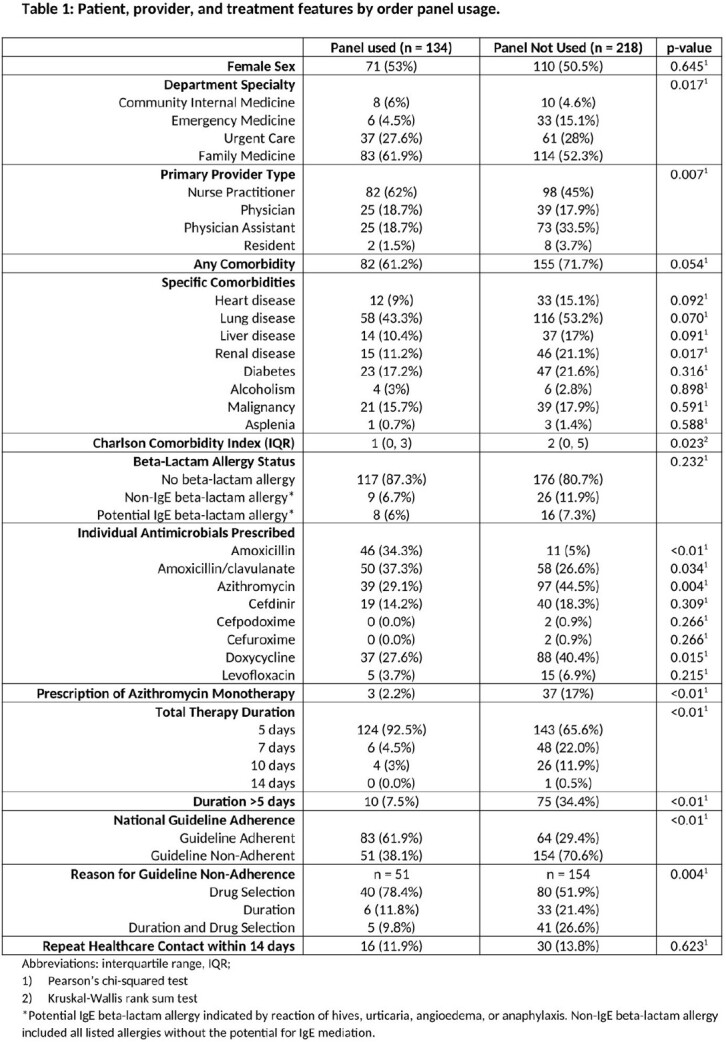

**Conclusion:**

Use of a pre-populated antimicrobial order panel in outpatients with CAP resulted in greater adherence to national guideline recommendations.

**Disclosures:**

**Paschalis Vergidis, MD, MSc**, AbbVie: Advisor/Consultant|Ansun: Grant/Research Support|Cidara: Grant/Research Support|Scynexis: Grant/Research Support

